# Improving hospital-based processes for effective implementation of Government funded health insurance schemes: evidence from early implementation of PM-JAY in India

**DOI:** 10.1186/s12913-021-07448-3

**Published:** 2022-01-15

**Authors:** Anurag Saxena, Mayur Trivedi, Zubin Cyrus Shroff, Manas Sharma

**Affiliations:** 1 Indian Institute of Public Health Gandhinagar, Opposite Airforce Head Quarters, Near Lekawada Bus Stop, Chiloda Road, Lekawada CRPF P.O, Gandhinagar, Gujarat India; 2 Alliance for Health Policy and Systems Research, World Health Organization, Geneva, Switzerland

**Keywords:** Hospital-based processes, PM-JAY, India, Health Insurance, Universal Health Coverage, Ayushman Bharat

## Abstract

**Background:**

Government-sponsored health insurance schemes (GSHIS) aim to improve access to and utilization of healthcare services and offer financial protection to the population. India’s Ayushman Bharat Pradhan Mantri Jan Arogya Yojana (PM-JAY) is one such GSHIS. This paper aims to understand how the processes put in place to manage hospital-based transactions, from the time a beneficiary arrives at the hospital to discharge are being implemented in PM-JAY and how to improve them to strengthen the scheme’s operation.

**Methods:**

Guidelines were reviewed for the processes associated with hospital-based transactions, namely, beneficiary authentication, treatment package selection, preauthorization, discharge, and claims payments. Across 14 hospitals in Gujarat and Madhya Pradesh states, the above-mentioned processes were observed, and using a semi-structured interview guide fifty-three respondents were interviewed. The study was carried out from March 2019 to August 2019.

**Results:**

Average turn-around time for claim reimbursement is two to six times higher than that proposed in guidelines and tender. As opposed to the guidelines, beneficiaries are incurring out-of-pocket expenditure while availing healthcare services. The training provided to the front-line workers is software-centric. Hospital-based processes are relatively more efficient in hospitals where frontline workers have a medical/paramedical/managerial background.

**Conclusions:**

There is a need to broaden capacity-building efforts from enabling frontline staff to operate the scheme’s IT platform to developing the technical, managerial, and leadership skills required for them. At the hospital level, an empowered frontline worker is the key to efficient hospital-based processes. There is a need to streamline back-end processes to eliminate the causes for delay in the processing of claim payment requests. For policymakers, the most important and urgent need is to reduce out-of-pocket expenses. To that end, there is a need to both revisit and streamline the existing guidelines and ensure adherence to the guidelines.

**Supplementary Information:**

The online version contains supplementary material available at 10.1186/s12913-021-07448-3.

## Background

In the context of low and middle-income countries, government-sponsored health insurance schemes (GSHIS) have in many instances been found to improve access and utilization of healthcare services, and reduce out-of-pocket expenditure [1–4]. In the literature, studies have also documented the challenges that are faced by beneficiaries and healthcare providers in GSHIS. From the beneficiary perspective, the challenges faced are in terms of lack of information, illegal fee collection, delays in the registration process, long waiting times, and poor perceived quality of healthcare services [5, 6]. For the healthcare providers challenges are in terms of delay in claims payments, increase in the workload for getting the claim processed [7]. Such challenges can reduce the attractiveness of GSHIS and may have a negative impact on the desired outcomes of the scheme. It is hence necessary for decision-makers to identify the challenges that are faced by healthcare providers and beneficiaries in a GSHIS and make an attempt to solve them.

Many of the challenges that are faced by the empanelled providers or hospitals and beneficiaries can be the outcome of the way in which processes to manage the operations of GSHIS are conceptualized and implemented. These processes determine the flow of information, money, and material in the context of a given GSHIS and cause deviations from desired outcomes. An understanding of the way these processes are designed and implemented and the effect they have on the empanelled hospitals and beneficiaries can highlight the changes that need to be made to strengthen the operations of GSHIS and overcome some of the challenges that are faced by the empanelled hospitals and intended beneficiaries.

In the existing literature, attempts have been made to study GSHIS in terms of their impact on access to and utilization of healthcare services, out-of-pocket expenditure, and determinants of participation in GSHIS [8–12]. However, there has been a lack of attempts to understand the way processes to manage the operations of a GSHIS are conceptualized and implemented and the effect they have on empanelled hospitals and beneficiaries. Such an understanding will help in informing the design and management of these processes. This paper aims to address this gap by analyzing the translation of operational guidelines into the processes associated with hospital-based transactions and understand how these processes function, and how to manage them to strengthen the operations of GSHIS. For doing so, this paper attempts to understand from the perspective of healthcare service providers (hospitals) the early implementation experience of the Ayushman Bharat Pradhan Mantri Jan Arogya Yojana (PM-JAY), one of the world’s largest government-funded health insurance schemes.

### Pradhan Mantri Jan Aarogya Yojana (PM-JAY)

Launched in September 2018, PM-JAY aims to provide insurance cover to 107.4 million poor and vulnerable families – i.e. up to 500 million Indians – for INR. 5,00,000 (USD 6666.67)^1^ per annum for all secondary and most tertiary care procedures of surgery, medical, and daycare treatments at public and empanelled private hospitals. In terms of implementation structure, PM-JAY has a three-tier implementation framework involving a central level entity, the National Health Authority (NHA), state-level entities called State Health Agencies (SHA), and a District level implementation unit. The NHA is responsible for the overall planning and implementation of the scheme and the central-state coordination. The SHA is a nodal agency for state-level implementation of the scheme. The District level implementation unit is responsible for coordinating operations at the local level. In each of the empanelled hospitals, the hospital nominates one of its medical doctors to act as a hospital-level nodal officer for the PM-JAY. Along with a nodal officer, each of the hospitals also deputes a desk-level staff called Ayushman Mitra (AM) to manage the operations, and liaise with patients, doctors, SHA, and hospital administration.

The operations of PM-JAY involve information exchange across diverse stakeholders –beneficiaries, AMs, hospitals, and insurer – over an IT-enabled platform. Three major components of the PM-JAY IT system are the Beneficiary Identification System (BIS), Hospital Transaction Management System (TMS), and Grievance Management System (GMS). The BIS is used to search the list of eligible beneficiaries to identify and register targeted individuals. The TMS is used to capture in-patient data at the hospital level for admission, treatment, and discharge, and share the data with the insurer for hospital claims and financial settlement. The GMS is for beneficiaries to register grievances and for NHA/ SHAs to respond to the grievances.

Under PM-JAY, empanelled hospitals are paid based on services provided, fixed rates for particular packages. As deemed suitable in a state by the SHA, those packages that are prone to abuse in the form of unnecessary procedures or fake surgeries are reserved for the public hospitals [13, 14]. These treatment/procedures are paid under PM-JAY only when availed by the beneficiaries in a public hospital and can be availed in a private hospital only after a referral from a public hospital. Those beneficiaries who avail these treatment/procedures in a private hospital without a referral from a public hospital need to pay the expenses on their own [15].

In PM-JAY, hospitals are important stakeholders that not only provide treatment but are also involved in real-time verification and authentication of beneficiaries, preauthorization for treatment, and coordination with the state agency for timely reimbursement of the claims. Under PM-JAY, clearly defined guidelines have been developed for each step of the hospital-based transactions. These include guidelines for preparatory activities for the empanelment of the hospital, and routine processes carried out for beneficiary authentication, medical treatment (also referred to as package under PM-JAY) selection, preauthorization, discharge, and claim reimbursement [16].

### Rationale for the study

While the guidelines are in place and capacity-building efforts are underway, there remains a gap in knowledge around the translation of guidelines into processes, how these processes are functioning, and how to manage them to strengthen the operations of PM-JAY. This research aims to address this gap in knowledge. The understanding of the translation of guidelines into processes and the way these processes are functioning will help in highlighting the changes that need to be made to strengthen the operations of GSHIS, this will in turn help to overcome some of the challenges that are faced by the empanelled hospitals and intended beneficiaries.

## Methods

This research was carried out with support from the WHO and in consultation with NHA. For finalizing the research method and study location, a meeting was carried out with the NHA team. In the meeting, the background of the study, research objectives, and research methods including the data analysis plan was presented. During the meeting, in consultation with the NHA, Gujarat and Madhya Pradesh (MP) states of India were selected as study locations. These states were selected as they have a difference in the experience of implementing GSHIS before the introduction of PMJAY in each of these states. Of these two states, Gujarat has a history of implementing a state government-sponsored health insurance scheme, namely, Mukhyamantri Amrutam Scheme.^2^ On the other hand, before PM-JAY, MP did not have any state government-funded health insurance scheme offering entitlement-based hospitalization coverage. Also, before PM-JAY, MP has limited experience of participating in a central government-managed health insurance scheme [17]. In Gujarat, PM-JAY is being implemented in a model wherein claims up to INR 50,000 (USD 666.67) are processed through an insurance company, and for claims more than INR 50,000 (USD 666.67), the insurance company makes the payment of INR 50,000 (USD 666.67), and amount over and above INR 50,000 (USD 666.67) is paid by the State Nodal Agency (known as ‘Trust’). In MP, the scheme is being implemented through a Trust wherein claims of all values are processed by the State Nodal Agency. For this study, within each state, seven hospitals were selected by the respective SHAs to ensure representation of public and private, and multi-specialty and super-specialty hospitals (Refer Table 1 for the distribution of hospitals).

**Table 1:** Details of hospitals and respondents

State	Ownership Type	Hospital type	Number of Hospitals	Interviewees^a^	Qualification of AM
Gujarat	Private	Multi-specialty	2	3 AM, 2 NO, 1 HCP, 2 Head	General Graduate, AYUSH Doctor^b^, Master in Hospital Administration
		Super-specialty	2	2 AM, 2 NO, 2 HCP, 2 Head	General Graduate
	Public	Multi-specialty	2	2 AM, 2 NO, 2 Head	General Graduate, AYUSH Doctor
		Super-specialty	1	3 AM, NO, Head	AYUSH Doctor
Madhya Pradesh	Private	Multi-speciality	2	2 AM, 2 NO, 2 HCP, 2 Head, 1 Finance Officer	General Graduate
		Super-specialty	2	2 AM, 2 NO, 2 HCPs, 2 Head, 1 Finance Officer	General Graduate
	Public	Multi-specialty	3	3 AM, 3 NO, 2 Head	2 Nurse, 1 Pharmacist

^a^*AM* Ayushman Mitra, *NO* Nodal Officer, *HCP* Healthcare Provider, *Head* Head of the hospital

^b^*AYUSH* A collective term used for Ayurveda, Yoga & Naturopathy, Unani, Siddha and Homoeopathy systems of medicine

To begin with, official guidelines developed by the NHA for each step of the processes to be carried out for hospital-based transactions were reviewed. The guideline describes the processes for hospital-based transactions under the following sub-headings: preparatory activities for the empanelment of the hospital, and routine processes carried out for beneficiary authentication, medical package selection, preauthorization, discharge, and claim reimbursement (Refer to Table 2 for the guidelines). Along with the review of guidelines, a semi-structured interview guide for interviewing stakeholders and a checklist of things to observe in each of the hospitals were prepared and shared with the WHO team. Based on the comments received from the WHO team, the interview guide and observation checklist was revised (Refer to Additional file 1 for interview and observation guide). For data collection, a letter of support for the study was obtained from the NHA. Subsequently, SHA and hospitals were contacted.

**Table 2:** Guidelines on processes for hospital transaction

Guidelines	Implementation status in Gujarat	Implementation status in Madhya Pradesh
**Preparatory Activities for State/ UT’s**		
Availability of requisite hardware, software, and allied infrastructure required for PM-JAY scheme activities	√	√
Medical Officer as Nodal Officer at EHCP^a^ for PMJAY has been nominated	Private hospitals (Except one in MP) had non-medical Nodal Officers	
Ensure appointment of Ayushman Mitra	√	√
Ensure that a dedicated helpdesk for PM-JAY at a prominent place	5 of 7	√
Availability of printed booklets at the helpdesk, which will be given to beneficiaries along with the PMJAY e-cards	Х	Х
State Health Agency (SHA) shall set up team(s) to handle hardware and basic software support, troubleshooting, etc.	√	√
Training of EHCP staff and Ayushman Mitras by the SHA/ Insurer	√	√
	(Training is software centric)	
**Process for Beneficiary identification, issuance of e-card, and transaction for service delivery**		
Operator/Ayushman Mitra identifies the beneficiary’s eligibility and registers patients	√ (Private hospitals choosing not to issue card)	√
TPIN (Telephonic Patient Identification Number) in case of emergency	No TPIN; Patients admitted and registered next day	
**Package Selection**		
Based on the diagnosis sheet provided by the doctor, the operator should be able to block the benefits package(s) using PM-JAY IT system	√	√
**Pre-authorization**		
Operator/Ayushman Mitra to initiate a request for pre-authorization to the insurer using PM-JAY IT system	√	√
The decision on the request by the insurer latest by 6 h. If not, the request deemed to be approved by default	3–4 h	Up 24 h
Insurance Company/ Trust will provide the reasons for rejection	√	√
The beneficiary or hospital can appeal through the grievance system	No grievance system. Hospitals re-apply with required documents	
**Balance Check, Treatment, Discharge and Claim Request**		
Based on the selection of package(s), the operator will check from the Central PM-JAY server if sufficient balance is available with the beneficiary to avail services.	Low balance not encountered as the scheme is still new	
The operator fills the online discharge summary form and the patient will be discharged	√	√
The beneficiary will be discharged with a discharge summary	√	√
**Claim Payments and Turn-around Time**		
The Trust/Insurer or the agency (Insurance Regulatory and Development Authority of India compliant only) appointed by it shall decide on the acceptance or rejection of any claim received from an EHCP. Any rejection notice issued by the Trust/Insurer or the agency to EHCP shall clearly state that rejection is subject to the EHCP’s right to appeal against the rejection of the claim.	All hospitals shared that the rate of claim rejection is very low. However, they reported that SHA or insurer do not convey a reason for the rejection	
The process in relation to claim shall be carried out in such a manner that its completion (Turn-around Time, TAT) shall be no longer than 15 calendar days (irrespective of the number of working days). For claims outside the State, a time of 30 calendar days will be provided.	Average TAT is around 90 days	

Source for guidelines: PMRSSM guidelines on processes for hospital transaction [16]

^a^*EHCP* Empanelled healthcare provider

All methods for the study were performed in accordance with the relevant guidelines and regulations. Ethical review for the study was conducted by the Institutional Ethical Committee of the University where the researchers are affiliated. Before conducting the interviews, informed consent was taken from the respondents. During field visits, an average of three days were spent in each hospital. In each of the hospitals, before starting the data collection, a brief meeting was conducted with the head of the hospital and key staff members wherein the background of the study was discussed with them, an assurance to protect the identity of the respondents was given to them, and any query they had about the study was answered. In each of the visited hospitals, it was planned to interview AM, one doctor, Nodal Officer, and the head of the hospital. In each of the hospitals, based on the discussions held during the initial meeting and availability of staff members during the days of the visit, names of those whom the research team could interview were shared by the hospital team. In some of the hospitals, name of more than one AM whom the research team can interview were shared. In such cases, all the AMs mentioned by the hospital team were interviewed. In case of any variation in responses of AMs in these hospitals, these were discussed with the respective Nodal Officer. The clarification by the Nodal Officer was considered final.

During the visit to hospitals, using the interview guide, in-depth interviews were carried out (Refer Table 1 for the number of interviews conducted and the profile of interviewees). The interviews were focused on identifying and mapping the steps that were carried out as a part of preparatory activities for the empanelment of the hospital, and hospital-based transactions, and the challenges and issues that are faced by the hospitals in implementing them. A total of fifty-three respondents were interviewed. Along with the interviews, a walk-through of the processes was carried out in all of the hospitals and in a few of them, informal interactions with the finance staff were also conducted. With due permission from the respondents, the interviews were audio-recorded and pictures in the hospitals were clicked. Along with the hospitals, interviews with the State Health Agency (SHA) official were also planned to gather their perspectives on the hospital-based processes. This was done with Gujarat SHA only, as an appointment with Madhya Pradesh SHA could not be made despite several attempts. During interaction with SHA in Gujarat, the hospital-based processes as getting followed in Gujarat were shared with the SHA. The sub-components of the claim payment process are beyond the hospital’s line of sight as they happen at the end of Insurer/Trust. The reasons for the issues faced by the hospitals in the claim payment process were shared by the SHA. As SHA in MP could not be interviewed, hence, the reasons for the issues faced by the hospitals in MP in the claim payment process cannot be enquired.

After visiting the hospital, all the interviews taken in that hospital were transcribed by one of the members of the research team, and a list of observations made in the hospital was compiled. These transcripts and observation lists were reviewed by the remaining members of the research team. After visiting all the hospitals in both the states, the transcripts of the interviews were analyzed manually to understand the steps that were carried in each of the hospitals in terms of preparatory activities for the empanelment of the hospital, and routine processes carried out for beneficiary authentication, medical package selection, preauthorization, discharge, and claim reimbursement. The description of the processes and process parameters provided in the standard official guideline by the NHA was used as the framework for analyzing the interviews and documenting the hospital-based processes followed in Gujarat and MP (Refer to Table 2). From the interviews, process maps were developed to understand the flow of information, money, and materials in each of the hospitals, and these process maps were then compared with the guidelines. For each of the processes described in the official guidelines, the interviews were analyzed to identify a) reasons by the hospitals for any deviation from the guidelines, b) challenges faced by the hospitals in implementing the processes, c) steps taken by them to overcome these challenges, and d) perceptions of changes required in policies and guidelines to smoothen day to day implementation. The findings from the interviews were corroborated with detailed non-participant observation of hospital staff involved in PM-JAY related processes and operations. The results of this study were then documented using the flow of processes provided in the standard official guideline as a template. The findings of this study were then subsequently discussed in a dissemination meeting with NHA and SHA officials. The entire process of conceptualizing the study, data collection, and analysis was carried out from March 2019 to August 2019.

## Results

**Fig. 1:**
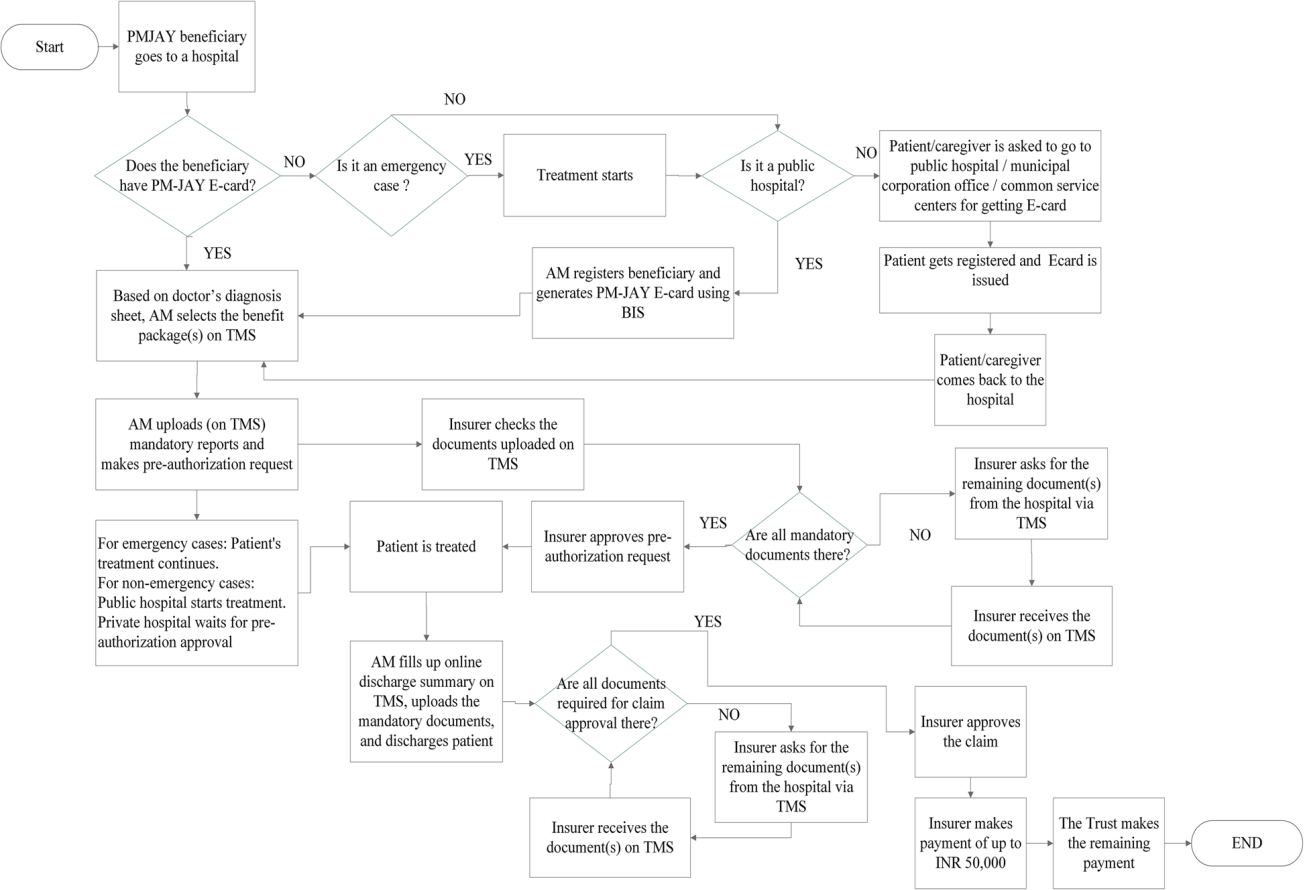
Process flow diagram for Gujarat

**Fig. 2:**
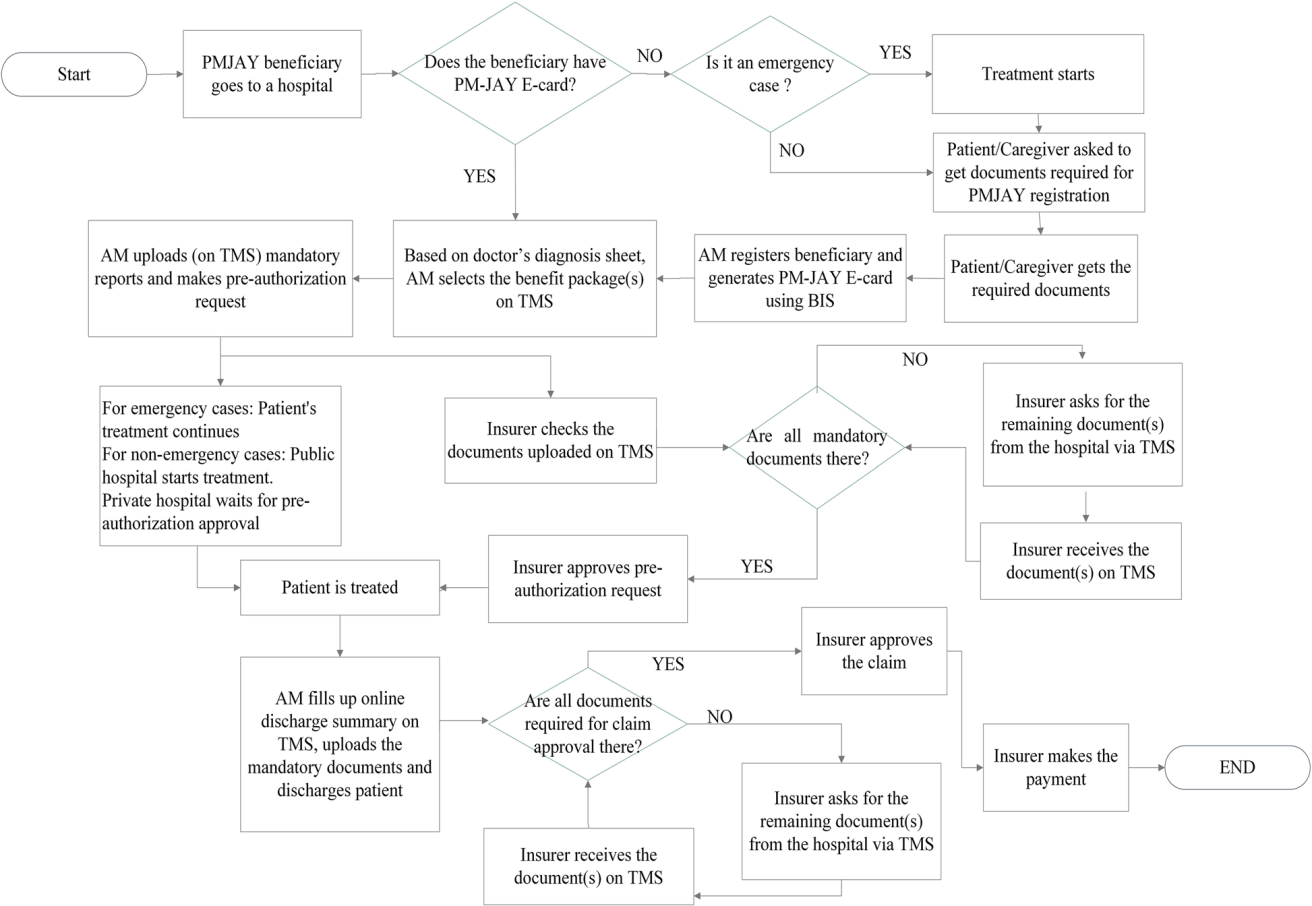
Process flow diagram for MP

The findings of the study are divided into subsections that correspond to the PM-JAY guidelines for hospital-based transactions. These sub-sections are preparatory activities for the empanelment of the hospital, and routine processes carried out for beneficiary authentication, medical package selection, preauthorization, discharge, and claim reimbursement (Refer to Table 2 for the guidelines for each of the sub-section). Each sub-section below provides insights from the interviews and non-participant observation of hospital staff. Figs. 1 and 2 show the process flow diagrams starting from the beneficiary authentication stage to the hospital claim settlement stage for Gujarat and MP respectively.

### Preparatory activities for the empanelment of the hospital

All the steps, except one about the availability of printed booklets at the helpdesk, described in the guideline under this sub-section were found to be implemented well in most hospitals in both the states. It was observed that all hospitals had hoarding and banners providing information about the scheme, however, no hospital had printed booklets for the patients.

All the hospitals had AMs in place, with differences in their qualifications. The AM in six out of fourteen hospitals were nurses or doctors of an alternative system of medicine. One of them also had a post-graduation in hospital administration (Refer to Table 1 for details on AM’s qualification).

For training of providers, especially that of the AM, all hospitals indicated receipt of training, however, they were of the opinion that the training was software-centric and lacked in content around building capacity in package selection, documentation, and other processes.

*The training provided to the AMs is for making them aware of the working of hardware and software parts… All the trainers knew computers but had no idea about packages and were not able to solve queries related to the packages.*

- Nodal Officer of a private hospital in Gujarat

### Beneficiary authentication

This involves the use of the BIS for beneficiary identification for issuance of PM-JAY e-card that enables the beneficiary to access care. Both public and private hospitals in MP register patients using BIS, but the private hospitals in Gujarat do not register beneficiaries and indicated that the patients are required to register themselves in the BIS at the public hospital / municipal corporation office / common service centers. Thus, in MP most of the PM-JAY beneficiaries reaching the empanelled hospitals for treatment do not experience a delay in the start of the treatment for want of a beneficiary card. In Gujarat, patients reaching private hospitals need to go to a nearby Government hospital/office for the card and then come back to the hospital for the treatment, thus causing a delay.

An area of deviation from the implementation of the guideline was regarding the use of the Telephonic Patient Identification Number (TPIN) in case of an emergency. As per the guideline, in case of emergency, a telephonic pre-authorization approval for the treatment can be taken by the hospital from the insurer wherein a pre-authorization code is communicated to the hospital over the telephone itself, and later using the code the case can be registered by the AM in the TMS. The system of TPIN was found to be non-existent in both states. However, the absence of TPIN did not appear to affect the admission of patients in case of an emergency. It was reported by the AMs that under PM-JAY, hospitals can register a patient up to five days after admission. Hence, in an emergency, patients are admitted and treated first and are registered later.

### Medical package selection

The AMs in all the hospitals were able to operate TMS for making a pre-authorization request (also referred to as blocking a package). The AMs were also able to scan and upload the documents required for getting pre-authorization approval in the TMS. This was relatively more efficient in the hospitals where the AMs had a medical/paramedical background. They were found to make hospital operations efficient by eliminating or substantially reducing the queries through a) understanding the doctor’s prescription and choosing the appropriate package to be blocked, and b) uploading the necessary medical reports required for the selected package at the first instance itself.

*Our hospital is doing well in the state, in preauthorization, claim submission, etc. Till now we have placed claims of more than INR 10 million (USD 0.13 million). We have been able to do this, because, we have put AMs from our technical staff, they know about medical terminology, medical procedures, coding, etc. hence are able to do a good job”*

– Head of a public hospital in Gujarat

### Preauthorization

Once the package is selected and blocked, a request is sent to the insurer for pre-authorization. As per the guidelines, the insurer has to either approve or reject the pre-authorization request within six hours of receiving the request, in case if the insurer fails to do so then after six hours the request is auto-approved. As opposed to the guideline, no hospitals indicated the system of auto-approval after six hours of requesting preauthorization approval. All hospitals in Gujarat reported an average time of three-four hours for pre-authorization approval, whereas private hospitals in MP reported that at times pre-authorizing approval took up to twenty-four hours. While private hospitals wait for approval to begin the treatment, public hospitals in both states initiate treatment. Hospitals indicated that during preauthorization they get queries from the insurer for more information, such as requests for detailed information from the treating doctor and/or investigation reports. Hospitals in MP indicated that at times they get queries even after six hours of submitting the pre-authorization request and at times these queries are not in alignment with treatment protocols. Such instances resulted in a delay in the initiation of the treatment.

*…Recently, in a case of Chronic Obstructive Pulmonary Disease (COPD), a query came where Operation Theater notes were asked. Now, COPD is a medical condition. We don’t know if the person raising the query has any medical knowledge or not.*

*–* AM (with a medical background) at a public hospital in MP

None of the hospitals reported any incidence of repeated rejection of the pre-authorization request. Certain hospitals, where the AMs were individuals with medical and managerial qualifications, were found to be innovatively using a) WhatsApp® for transfer of patient details, bedside photos and other work details, b) Mobile phone calls for coordination with patients and relatives, and c) ward boys and other staff for sending patient files to and from the TMS counters. These measures reduced patient movement and improved the efficiency of the work.

It was shared by the hospitals that the TMS does not provide a procedure-wise list of documents as hints/pointers at the submission window, something that can reduce or eliminate the chance of missing out on essential documents for pre-authorization and claim settlement request. One of the hospitals in MP has developed software to meet this felt need to reduce the time for approvals and claim settlement.

*"TMS software doesn't provide us with prompts on the submission window. We used to have many incomplete submissions and it used to take significant time for claim settlement. So, we designed a software for managing our submissions…..Since we started using the software, there have been fewer queries from incomplete document submission. It saves our time and ensures timely settlement of claims."*

- Senior AM at the hospital

### Discharge

Guidelines for discharge state that the operator should fill the online discharge summary form and the patient should be discharged with a discharge summary. This was being followed in all the hospitals. In case the PM-JAY portal was running slow or there was a higher number of patients to discharge, the AM scanned and stored all the documents in his/her computer, and patients were physically discharged from the hospital. Later, these scanned documents were uploaded along with the discharge request on the PM-JAY portal.

During discharge, one of the main concerns of the hospitals and patients is payment. Almost all hospitals raised concerns about the rates and content of various packages and hoped that they will be revised soon through a consultative process. Although the PM-JAY offers free treatment to patients, certain private hospitals indicated that they charged their patients. In some cases these were lump-sum payments to be paid at the hospital counters and in some others, these were expenses incurred in purchasing certain medicines or consumables. The payments were asked from the beneficiaries in three instances, namely, a) to cover pre-hospitalization expenses for pre-operative investigations, b) in case patients needed multimodal treatment then they were asked to pay for medical packages that were reserved for public hospitals but were availed in a private facility, and c) to part pay the difference between the PM-JAY package reimbursement and the charge for the provider’s preferred treatment (e.g. choice of medicine or implants). Hospitals insist on payment of pre-operative diagnostic procedures as there is a possibility that the patient may not be hospitalized based on the negative results, leading to their claim not being entitled for reimbursement under PM-JAY. Certain private hospitals insist that patients get these done at public hospitals. Some hospitals indicated that once patients are admitted, the hospital pays beneficiaries back the cost of pre-operative investigations. In MP, 472 packages (mostly non-surgical procedures) are restricted for government hospitals since the launch of PM-JAY. These procedures are not covered in empanelled private hospitals. So, the patients needing multimodal treatment (using more than one package) when admitted into a private hospital, may end up paying for packages reserved for public hospitals, without necessarily knowing this at the time of admission.

*… Breast cancer is one of the common cancer here. Cancer treatment uses multimodality treatment and requires teamwork from surgical, medical, and radiation oncology teams. Surgery for this condition is covered under PM-JAY for private hospitals like ours, but the chemotherapy is reserved for the government hospital. When we tell this to the patient, they refuse to go to the government hospital, which by the way doesn’t even have a medical oncologist. This way the patients have to pay for services that are supposed to be covered.*

*-* Healthcare provider at a private hospital in Madhya Pradesh

Private hospitals also mentioned instances of patient-provider disputes when at the time of discharge beneficiaries are asked to make payment for packages that are reserved for public hospitals.

### Claim reimbursement

PM-JAY guidelines for hospital-based transactions indicate that the turn-around time for claim payment should be fifteen days for within-state and thirty days for interstate claims. According to the tender documents of PM-JAY hospital empanelment in Gujarat, the turnaround time for claim payment should be forty-five days. However, hospitals in both states reported an average turn-around time of more than ninety days for full payment of their claims. The reasons for the delay are different in the two states.

In the hybrid model of Gujarat, the insurance company first makes the payment up to INR. 50,000 (USD 666.67) and then passes on the details of the payment to the Trust. The Trust then makes the remaining payment. The first tranche of claim is processed within forty-five days, but the remaining payment by the Trust gets delayed. Thus, hospitals in Gujarat have indicated a long turnaround time in full payment. Also, the hospitals in Gujarat indicated that they receive a lump sum amount in their bank account without the corresponding information on the PM-JAY system linking the amount to specific claims, thus, making it difficult for them to match the claims against the payment received.

*We do not receive payment information in a proper format. So it becomes very difficult for us to verify the amount with the number of patients and track their payment details... We know that a certain proportion of total pending funds have been paid, but cannot tally them.*

- Nodal Officer of a private hospital in Gujarat

In MP, hospitals indicated that insurer often makes repeated queries very late in the claim settlement stage. Some hospitals also expressed dissatisfaction regarding the full or partial rejection of claims by the insurer without being given clear reasons for the rejection, and the lack of redressal thereof.

*…for a case recently, a query asking for a post-operative MRI came after 3 months. Luckily, for my satisfaction, I had done a post-operative MRI and I was able to submit a report. Otherwise, where would we have searched for the patient?*

- Healthcare provider at a private hospital in MP

### The positive externality of PMJAY

In addition to the above-mentioned findings, some positive externalities of the PM-JAY were also found. These are:

1. Approval of pre-authorization request and claim acceptance on the PM-JAY portal requires doctors’ notes, test results, and a general level of detailed documentation. During the interviews, hospitals indicated that as a result of queries raised during pre-authorization and claim processing, doctors are becoming aware of the detail with which they should be noting their observations, prescriptions, etc. in patient’s case papers. As per the hospitals, over time this has led to improvement in case documentation by doctors.
2. Hospitals run by NGOs/charitable organizations typically serve the economically weaker sections of the society. Several of these hospitals have empanelled themselves and are receiving a bulk of their operating expenditure through PM-JAY. Hospitals indicated that this enables them to invest in capacity-building efforts and undertake capital expenditures from funds generated by them through other sources. With the hospital’s operating expenditure taken care of by PM-JAY, it was also shared that these organizations are also able to invest in other social causes such as education, skill development of the marginalized section of the society.

## Discussion

This research highlights the way guidelines pertaining to the hospital-based transactions under the PMJAY scheme are implemented at the level of empanelled hospitals. The results of this study provide details on how these processes are functioning and the effect they have on empanelled hospitals and beneficiaries.

The results of this study highlight that all the empanelled hospitals have PM-JAY hoarding and banners. However, none of them have printed booklets. In the context of Rashtriya Swasthya Bima Yojana (RSBY), an earlier GSHIS in India, the booklet provided to the beneficiaries at the time of registration had a list of empanelled hospitals, availability of benefits/entitlement, and the details of contact person(s) in case of need [18]. This kind of booklet can act as a source of information for the beneficiaries and as a citizen charter. In the absence of such booklets in PM-JAY, beneficiaries have only the hospitals to rely on for the information. This kind of booklet can also act as a common document for the hospitals and beneficiaries to fall back in case of any disagreement.

The results of this study highlight that in both Gujarat and MP, hospitals are not using the TPIN process. However, this has no impact on the admission of patients in case of a medical emergency. This suggests that there is a scope for revisiting the guidelines and simplifying the processes without having any negative impact on the outcomes of the PM-JAY scheme.

Under PM-JAY, capacity-building efforts are currently aimed at building AM’s capacity to operate PM-JAY’s IT platform. The findings of this study show that these efforts are achieving their intended purpose and all the AMs are familiar with the features of the software and can operate the software. PM-JAY guidelines for hospital-based transactions specify that on doctor’s prescription AM will book the package. In practice, it was found that AMs with knowledge about medical terminology, medical procedures, and package-related documentation are better equipped to support doctors in carrying out package-related documentation and in resolving insurer’s queries about the package and its documentation. However, in the absence of capacity-building efforts in medical terminology, medical procedures, and package-related documentation, the AMs - especially those from a non-medical background - are facing difficulties in supporting doctors in carrying out package-related documentation and in resolving insurer’s queries related to the package. Hence, there is a need to broaden the scope of capacity-building efforts to provide an understanding of medical terminology, medical procedures, and package-related documentation. In the existing literature, it has been suggested that frontline workers play a crucial role in the implementation of public policies [19–21]. For the success of PM-JAY, it is crucial to focus capacity-building efforts on the technical, managerial, and leadership skills of AMs.

The findings of this study highlight that nurses or doctors trained in alternative systems of medicine and managerially qualified AMs are able to complete hospital-based transactions efficiently by using a) WhatsApp® for transfer of patient-related data, b) Mobile phone calls for coordination with patients and relatives, and c) hospital staff for sending patient files to and from the TMS counters. At the level of hospitals, encouragement can be given to the AMs to develop and implement contextually relevant practices that increase the efficiency and security of hospital-based processes.

The results of this study indicate that PM-JAY beneficiaries in both states experience a delay in the start of medical treatment during hospitalization events. In Gujarat, PM-JAY beneficiaries experience a delay in the start of treatment for want of beneficiary card. Patients reaching private hospitals in Gujarat are asked to go to a nearby Government hospital/office to get PM-JAY card and then return to the hospital for treatment. This practice may have its roots in the way guidelines for the implementation of the earlier government-sponsored health insurance scheme, Mukhyamantri Amrutam, were designed. Under the Mukhyamantri Amrutam scheme, beneficiaries were registered only at a public hospital / municipal corporation office / common service centers, and only after getting registered beneficiaries were able to avail treatment [22]. In MP, PM-JAY beneficiaries experience a delay in the start of treatment due to the delay in pre-authorization request approval. It was reported by the hospitals in MP that at times pre-authorizing approval took up to twenty-four hours and at times queries raised during the pre-authorization phase are not in alignment with treatment protocols. With PM-JAY, using the principles of business process re-design [23], there is an opportunity for Gujarat and MP to evaluate the steps in the hospital-based processes and streamline the processes to ensure that the beneficiaries get the required treatment without any delay.

The average turn-around time for claim payment to the hospitals in both Gujarat and MP is many times more than the turn-around time mentioned in the PM-JAY guideline. Delay in the making of payment to the empanelled hospitals has also been reported in other insurance schemes in Indian and other contexts [24, 25] and has been found to be associated with negative behavior by healthcare providers towards the insured patients [26]. These findings indicate a need to streamline the back-end processes of PM-JAY to eliminate the causes of these delays. For example, in the case of Gujarat, the possibility of parallel processing of claims by both the Insurance agency and the State Nodal Agency needs to be explored wherein both insurance agency and State Nodal Agency pay their part of the claim and the claim as a whole gets settled within the stipulated time duration.

Patients availing treatment under PM-JAY are not required to make any payment to healthcare service providers, however, the results of this study highlight that beneficiaries in private hospitals are asked to make payments by healthcare service providers. Similar to the findings of the present study, in the context of earlier government-sponsored health insurance schemes in India, beneficiaries were asked to make payments by healthcare service providers [8, 9, 11, 27].

During the study, almost all hospitals raised concerns about low package rates and hoped that they will be revised soon through a consultative process. A similar observation has been made in a study carried out by the Federation of Indian Chambers of Commerce and Industry on the cost of procedures and rates offered under PM-JAY wherein the report concluded that there is a need for an upward revision in the rate of packages [28]. The low package rates in PM-JAY may be dissuading some healthcare service providers from enrolling themselves in the scheme and at the same time, it may be making some of the enrolled private hospitals selective about treating patients for certain medical conditions for which the package rates are relatively optimal while referring the remaining patients to other service providers.

The information exchange over the IT-enabled platform constitutes the backbone of PM-JAY’s operations. The results of this study highlight that there is scope for improvement in the TMS component of the IT-enabled platform wherein, at the submission window for a procedure selected by the operator, the list of documents that need to be submitted for pre-authorization and claim settlement could be provided to reduce or eliminate the chance of missing out on submitting one or more essential documents. This will help in reducing the time taken to complete the pre-authorization and claim settlement process.

The results of this study highlight that there is a difference in PM-JAY processes followed in the two states. In India, health is a state government subject. For PM-JAY, states have the flexibility to implement the processes in a manner deemed suitable by them. In PM-JAY, it has been observed that underdeveloped states and districts have verified a smaller proportion of their eligible population, empaneled few hospitals, and have a lower number of claims and claim volume [29, 30]. One of the main reasons that have been highlighted for such a variation is the difference in the capacity of sub-national governments to conceptualize and implement health policy and program tools [29, 31]. For the successful implementation of PM-JAY, there is a need for the Central and State government to collaborate for developing a quality assurance mechanism and enhance the capacities needed for the faithful implementation of the program.

An attempt to understand the processes carried out in the context of a GSHIS should ideally include the perspectives of both hospitals as providers and patients as consumers of healthcare services. The focus of this research work was to understand from the perspective of healthcare service providers (hospitals) the early implementation experience of PM-JAY. By focusing on hospitals only, this study has not been able to triangulate the obtained information with the patient’s perspective. In future studies, there is a need to focus on patients as consumers of healthcare services and bring their perspectives in the discussion on improving PM-JAY processes.

## Conclusions

This research contributes to the existing literature by shedding light on how operational guidelines are getting translated into processes associated with hospital-based transactions carried out under PM-JAY. The results of this study highlight the way these processes influence empanelled hospitals and beneficiaries and the changes that need to be made to strengthen PM-JAY operations. PM-JAY is a recent initiative and so the literature on it is limited. This study contributes to this thin literature base and offers suggestions to hospitals, program managers, and policy planners to strengthen PM-JAY operations.

At the hospital level, an empowered and informed AM is key to efficient hospital-based processes. AMs can be empowered through a) recruitment of personnel with clinical and hospital administration backgrounds, and b) active and ongoing capacity-building efforts of existing AMs. In hospitals, the use of phone calls and mobile-phone-based instant messaging technology for data transfer and coordination is a local practice that brought efficiency. However, there is a need to examine the privacy implications of sharing this data over private phones. Hospital authorities may encourage the identification, exploration, and development of local operational level practices for patient-centric, efficient, and secure processes for enhanced user satisfaction.

At the program management level, there is a need to introduce refresher training to identify and encourage operation-level practices that make processes efficient and secure, and learn from the analysis of claim-related queries. There is also a need to evaluate the steps in the hospital-based processes and streamline the processes to eliminate delays experienced by patients and empanelled hospitals. In PM-JAY, multiple departments are involved. Any process redesign effort will affect the work carried out by the involved departments. Getting the involved departments on board for redesigning and implementing the new processes may require looking at the entire exercise from the change management perspective. For such an initiative, explicit support from top management at NHA and SHA will be needed. Also, existing staff members at NHA and SHA who are trained in management science and know the existing process may become useful resources in this change management initiative.

For policy-makers, there is an immediate need to focus on causes of out-of-pocket payments by the beneficiaries. This study brings supply-side information in the form of constraints in terms of package design and restrictions for private providers. To reduce out-of-pocket payment for hospitalization under PM-JAY, there is a need for strict implementation of existing guidelines, re-looking at the division of packages into the categories of those restricted and not restricted for government hospitals, and a need to develop standard treatment guidelines through a consultative process to reduce the divergence between PM-JAY package specifications and provider’s preference for treatment. The reservation of packages for public hospitals has an impact on access to healthcare services wherein beneficiaries are not able to avail needed services at one place and end up making out-of-pocket payments. While developing standard treatment guidelines, it also needs to be acknowledged that even with standard treatment guidelines there will always be some divergence between PM-JAY package specifications and provider’s preference both due to providers wanting to provide what they think is the best as well as the underlying profit incentive for healthcare providers. Finally, there is a need to review and revise package rates, such that these are feasible for the program to pay as well as attractive enough for providers to want to provide services to PM-JAY beneficiaries.

## Supplementary Information

**Additional file 1.**

## Data Availability

The data used in the study belongs to the World Health Organization (WHO), which has restricted its sharing. Data are however available from the corresponding author upon reasonable request after seeking permission from the World Health Organization.

## Abbreviations

AM: Ayushman Mitra
BIS: Beneficiary Identification System
COPD: Chronic Obstructive Pulmonary Disease
GMS: Grievance Management System
GSHIS: Government-sponsored health insurance schemes
MP: Madhya Pradesh
NHA: National Health Authority
PM-JAY: Ayushman Bharat Pradhan Mantri Jan Arogya Yojana
SHA: State Health Agency
TMS: Transaction Management System
TPIN: Telephonic Patient Identification Number
WHO: World Health Organization

## Footnotes

1 1 USD = 75 INR

2
http://www.magujarat.com/
