# Supplementary material for: Improving hospital-based processes for effective implementation of Government funded health insurance schemes: evidence from early implementation of PM-JAY in India
Source: BMC Health Serv Res. 2022 Jan 15;22:73. doi: 10.1186/s12913-021-07448-3 (PMC8760668; doi:10.1186/s12913-021-07448-3)
Supplement: Supplementary file 1 — Additional file 1. [file 12913_2021_7448_MOESM1_ESM.docx]

**Appendix A**

1. **Interview guide for Ayushman Mitra and Nodal Officer**
2. Tell us about yourself, including your qualification, work experience, etc.
3. Explain your work in a step-wise manner
4. Have you undergone training for PM-JAY work?
   1. Duration of training
   2. Frequency
   3. Utility of training
   4. Suggestions for improvement in the content and delivery of training
5. Explain approaches to and issues with
   1. Beneficiary identification, authentication, and enrollment
   2. Package selection and blocking
   3. Preauthorization
   4. Discharge
   5. Payment made by the patients (if any)
   6. Claim settlement by the insurer
   7. Information exchange over PM-JAY IT systems
6. Most common challenges faced in dealing with
   1. Patients and their relatives
   2. Insurer
   3. IT hardware, software, and allied infrastructure
   4. Hospital staff
7. Any other important feedback/inputs for improving PM-JAY operations?
8. **Interview guide for Doctor**
9. Tell us about yourself including your qualification, work experience, etc.
10. Explain your work in a step-wise manner
11. What do you know about PM-JAY scheme? What processes are related to this scheme in your hospital?
12. Were you given any orientation about PM-JAY before/after your hospital got empanelled in this scheme?
13. Explain approaches to and issues with
    1. Package selection and blocking
    2. Preauthorization
    3. Discharge
    4. Payment made by the patients (if any)
14. What are the issues with the processes of PMJAY implementation?
15. What are your suggestions to address these issues?
16. Any other important feedback/inputs for improving PM-JAY operations?
17. **Interview guide for hospital head**
18. Tell us about your experience of working with PMJAY
19. What were the considerations behind joining PMJAY?
20. How PMJAY has changed the hospital (clientele, magnitude, and revenue)?
21. Please share positive and negative aspects of PMJAY’s processes.
22. What are your suggestions for improving hospital-based processes under PMJAY?
    1. How to make them more patient-friendly?
    2. How to make them more hospital-friendly?
23. Any other important feedback/inputs?
24. **Observation Guide**
25. Helpdesk: Exclusive or common desk for multiple works
26. Helpdesk location: Prominent or isolated, functionality
27. Information, Education, and Communication material availability
28. Signages in hospital about PMJAY
29. Steps and information exchange during
    1. Enrollment and registration
       1. Procedure followed by AM and beneficiaries for enrollment and registration
    2. Package selection, and blocking
       1. Format followed by doctor to communicate the information about package to AM
       2. Procedure followed by AM to block the package (including interaction with the patient).
    3. Preauthorization and admission
       1. Interaction of AM and Insurer/Trust through IT system
       2. Interaction of AM and patient about the outcomes of preauthorization request
       3. Issues around pre-authorization rejection viz. reasons, Interaction of AM and patients about reasons, patient education about grievance redressal
    4. Discharge
       1. Procedures followed by AM to discharge patients from the hospital, and details of documents uploaded on PM-JAY IT system
       2. Interaction of AM and patients regarding blocking post-hospitalization procedures and expenses
    5. Any other process/step/interaction deemed important
